# Size matters: implications of the loss of large individuals for ecosystem function

**DOI:** 10.1038/srep02646

**Published:** 2013-09-12

**Authors:** Alf Norkko, Anna Villnäs, Joanna Norkko, Sebastian Valanko, Conrad Pilditch

**Affiliations:** 1 Tvärminne Zoological Station, University of Helsinki, FI-10900 Hanko, Finland; 2 Marine Research Centre, Finnish Environment Institute, PO Box 140, FI-00251 Helsinki, Finland; 3 Department of Biological Science, University of Waikato, Private Bag 3105, Hamilton, New Zealand

## Abstract

Size is a fundamental organismal trait and an important driver of ecosystem functions. Although large individuals may dominate some functions and provide important habitat structuring effects, intra-specific body size effects are rarely investigated in the context of BEF relationships. We used an *in situ* density manipulation experiment to explore the contribution of large, deep-burrowing bivalves to oxygen and nutrient fluxes across the sediment-water interface. By manipulating bivalve size structure through the removal of large individuals, we held species identity constant, but altered the trait characteristics of the community. The number of large bivalves was the best predictor of ecosystem functioning. Our results highlight that (a) accounting for body size provides important insights into the mechanisms underpinning biodiversity effects on ecosystem function, and (b) if local disturbances are recurrent, preventing individuals from reaching large sizes, the contribution of large adults may be lost, with largely unknown implications for ecosystem functionality.

There is clear evidence that losses in biodiversity reduce the efficiency of ecosystem functions, including productivity and nutrient cycling^1^, but the actual mechanisms that underpin the positive biodiversity-ecosystem function (BEF) relationships remain an area of intense research^2^,^3^. A few species (with unique traits) may in fact dominate certain ecosystem processes^4^,^5^,^6^, and recent meta-analyses have indeed shown that species identity effects may be as important as richness effects *per se*^7^. Improving the mechanistic understanding of BEF relationships and allow prediction of the magnitude of change in ecosystem function following the loss of particular traits^2^,^8^ thus depends on the nature of the ecosystem function(s) of interest and trait composition not only across the resident community but also within species. Trait-based approaches have, however, largely focused on assigning traits to species rather than individuals^9^. Hence, while interspecific differences in trait composition and the subsequent functional contribution to ecosystem processes have been acknowledged, differences in intra-specific trait characteristics are rarely addressed^10^ (but see e.g.^11^,^12^,^13^).

Body mass is a fundamental organism trait that affects metabolic rate, energy demand and uptake rate^9^,^14^,^15^, and is an important characteristic of overall population and community structure through density-mass allometric relationships^14^. Even though high numbers of small individuals may dominate specific ecosystem functions through rapid turnover rates, the fewer large individuals may dominate other functions and provide important habitat structuring effects^16^. Particularly where ecosystem functions relate to the generation of biogenic habitat or organisms mediation of the nature and flux of energy and matter, size matters. This is potentially very important in marine sediments where large organisms can be expected to displace more sediment, pump more water and create stronger pore-water pressure gradients^17^. These are features known to affect major ecosystem functions such as nutrient and organic matter processing. While biomass has been recognized (or controlled for in partition experiments) as an important driver in several BEF studies^18^,^19^, body size effects are rarely investigated, particularly within species^9^,^20^. Because of the implications for mass-specific metabolic rates, it is important to know how biomass is distributed in an assemblage^20^,^21^. Importantly, an organism's contribution to ecosystem processes may change through ontogeny, but this is rarely considered in BEF analyses.

Common species are often the drivers of ecosystem processes. In marine soft-sediment habitats, large organisms that bioturbate (reshuffle and irrigate sediments) or suspension feed, can have dominant effects on nutrient regeneration and productivity that overshadow species diversity effects^22^,^23^. Bivalves, for example, can play pivotal roles in ecosystem functioning through their impact on benthic-pelagic coupling^24^,^25^, nutrient regeneration^26^ and their facilitation of surrounding communities^27^. Many bivalves are long-lived, grow to comparatively large sizes and dominate overall assemblage biomass, and can therefore serve as foundation species^28^. Large size and longevity, however, are both traits that make such species prone to extinction^29^,^30^. Further, species losses to disturbance are rarely random^31^ and large organisms are often vulnerable. The life-stages of individual species differ in their potential recovery following disturbance^32^. Bivalves have decreasinging mobility with increasing size and it is common for the small early life-stages to dominate recovery, while adult stages take considerable time to establish through growth. While complete extinctions of regional species pools are comparatively rare, compositional changes and reductions in abundance and biomass in the degradation process are common, so that recovering populations, while contributing to species richness contribute little to ecosystem function^33^. Increased mortality of one species to below its ecologically effective population size (EEP), while not making this species go extinct, may indeed have functional effects resulting in the extinction of other species instead^34^.

In marine systems very little attention has been directed towards changes in ecosystem function in the community assembly process following disturbance (but see^35^). Importantly, while species-abundance patterns may exhibit comparatively fast recovery^36^, communities may take considerable time to develop populations with undisturbed demographic characteristics^32^. Hence, if local disturbances are recurrent the contribution of large adult stages may be lost, with largely unknown implications for ecosystem functionality.

In the Baltic Sea, structural and functional biodiversity is naturally reduced due to low salinity, and the critical role of the few functional groups is apparent as losses of any species may entail a loss of the only representative of a function, such as suspension feeding^37^,^38^. It thus provides an ideal environment for empirical testing of key traits for ecosystem function. In addition very few benthic species in the Baltic Sea are long-lived or large, i.e. with traits that are predicted to have important influences on ecosystem function. The shallow soft-sediment communities are typically comprised of only a handful of species; the Baltic clam *Macoma balthica* and the soft-shell clam *Mya arenaria* typically make up an average of 15% of total community abundance and 75%, or more, of community biomass. Observations from recent manipulative field experiments in subtidal soft-sediment habitats suggest that community assembly processes following disturbance may result in substantial transient dominance shifts, with relatively quick recovery in terms of both species numbers and abundances^36^. Nevertheless, our observations also suggest that the recovery of mature and large-sized components of the bivalve populations may take considerable time (several years).

We conducted a field experiment to test the overall hypothesis that large adult bivalves are foundation species in soft-sediment communities with profound influences on ecosystem function. After disturbance it takes a long time for these adult bivalves to re-establish. Our prediction was that the contribution of bivalves to ecosystem function would mirror their relative dominance in terms of biomass. We tested this prediction by conducting an *in situ* density manipulation experiment where we (1) disturbed a community to eliminate all fauna to initiate a community assembly process where species-abundance patterns would have recovered (i.e. after 12 mo), but where large, mature life-stages would be lacking, and (2) seeded large individuals of bivalves (*Macoma* and *Mya*) to undisturbed control communities to obtain elevated densities (still within their natural range). We then incubated the sediment *in situ* to examine the contribution of deep-burrowing adult stages of large bivalves to measures of ecosystem functioning: ammonium and phosphate fluxes at the sediment-water interface, and community respiration. These measures are key ecosystem functions in soft-sediment habitats. We demonstrate that body-size is a key organism trait with important implications for understanding BEF relationships.

## Results

The two different sampling occasions were combined in our analysis of both macrofauna and fluxes to increase replication of our study. Bottom water temperature was 19°C at T1 and 13°C at T2. Water column concentrations of oxygen and nutrients differed slightly between T1 and T2 (O_2_: 9.43 ± 0.07 *vs.* 9.63 ± 0.03 mg l^−1^, PO_4_^3−^: 0.22 ± 0.01 *vs.* 0.46 ± 0.08 μmol l^−1^ and NH_4_^+^: 0.24 ± 0.05 *vs.* 0.30 ± 0.10 μmol l^−1^, respectively). We also observed variability in macrofauna community structure between sampling occasions. This variability, however, merely added strength of inference to our findings.

### Treatment effects on benthic community structure

**Figure 1:**
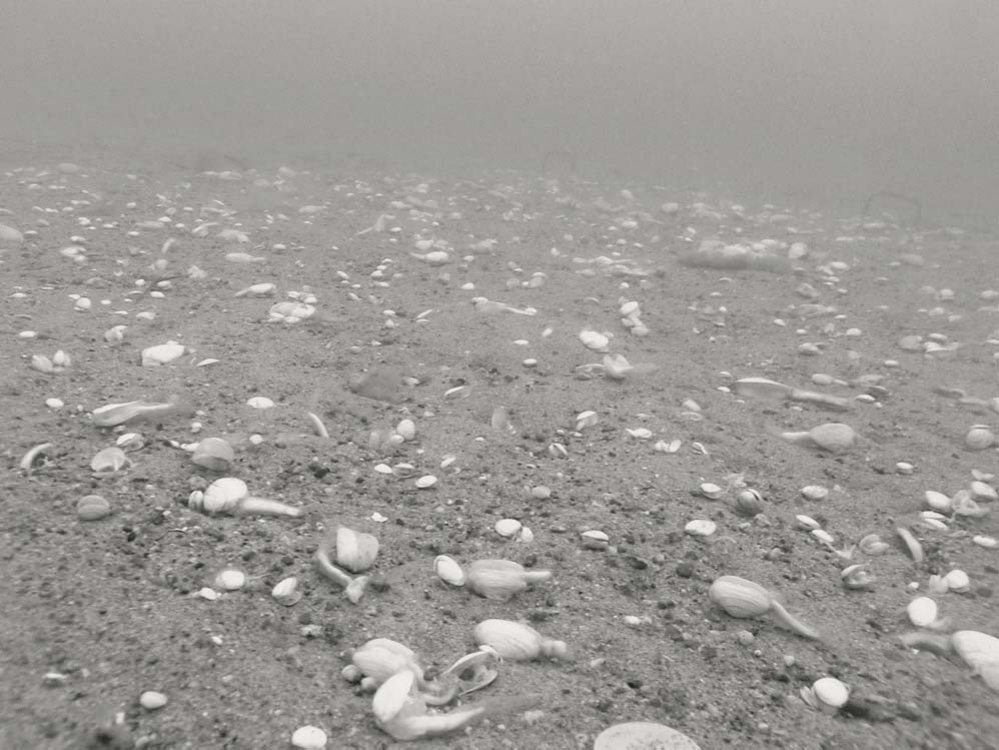
Dead adult bivalves on the sediment surface after a hypoxic disturbance event, which resulted in major changes in ecosystem function. The bivalves *Mya arenaria* and *Macoma balthica* are comparatively long-lived and the mature stages may take 5–10 years to re-establish after disturbance.

After the experimental disturbance (Fig. 1), the manipulated plots were left to recover for a year. After 12 months we observed no differences in diversity and the average numbers of taxa were more or less identical across treatments (Table 1).There were, however, differences in total community abundance and biomass, and in the distribution of bivalves (Table 1). Interestingly, the highest community abundance values were observed in the disturbed plots (D), which was also the case for bivalves in general and *Macoma* in particular. This may seem counterintuitive as bivalves were added to the elevated plots (E); however these differences could be explained by higher densities of post-settlement juvenile bivalves and polychaetes in plots with a disturbance history. Nevertheless, differences in total biomass and bivalve biomass were very clear, with the lowest biomasses observed in the recovering community (D) and the highest in the treatment to which bivalves had been added (E), with biomass values ranging from 56 to 460 g wwt m^−2^ (Table 1).

**Figure 2:**
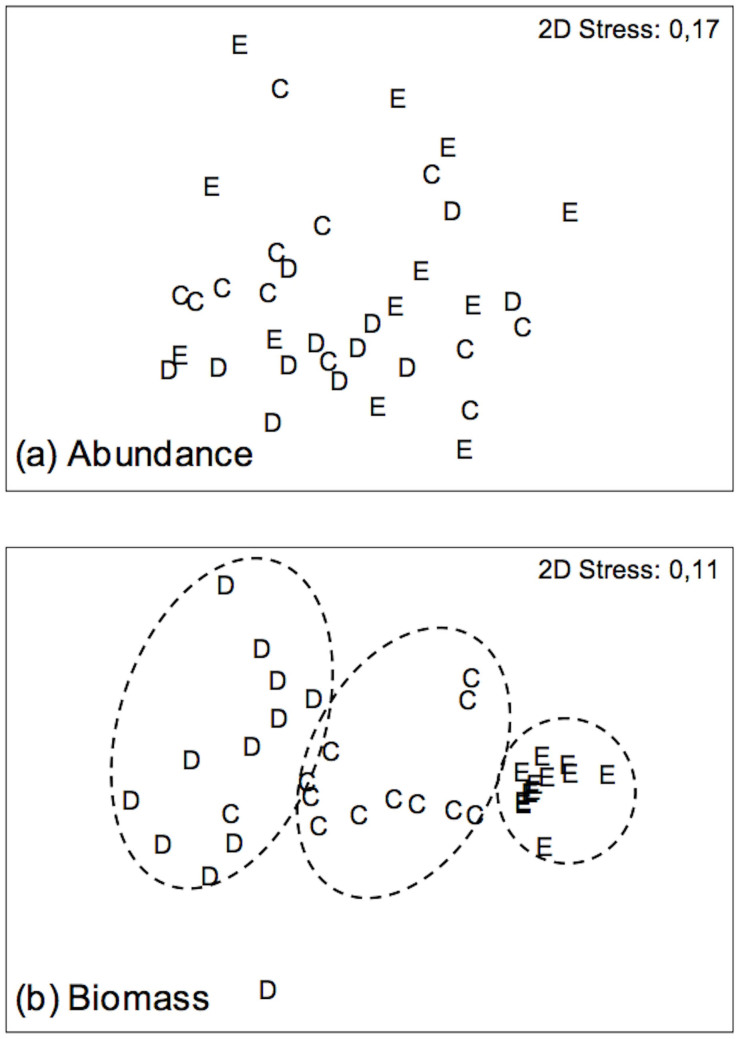
Multidimensional scaling analysis of community (a) abundance and (b) biomass. C = control, D = disturbed, E = elevated.

Multivariate analyses showed that overall community structure was not different between treatments for abundance, as no clear groupings were detected (Fig. 2a). In contrast, patterns of community structure in terms of biomass were distinctly different between treatments, forming clear groupings and a gradient from D, C to E (Fig. 2b). Interestingly the disturbed plots (D) exhibited the largest variability between replicates. The multivariate PERMANOVA analysis confirmed the significance of these patterns and showed that abundance variations indeed were non-significant (Pseudo-F = 1.53, p = 0.126), while groupings in biomass (Fig. 2b) were highly significant overall (Pseudo-F = 14.68, p = 0.001) and also between all treatments (Table 2).

The SIMPER analyses identified bivalves as contributing most to differences between sample clusters observed for biomass. The average group dissimilarity between C and D was 73% and the bivalves contributed > 60% of this difference. The average group dissimilarity between C and E was 72% and here bivalves (*Macoma* and *Mya*) contributed 94% of the difference between treatments. Overall community dissimilarity between D and E was, as expected, the largest at 93% and also here bivalves contributed most to the difference, 90%. Other common taxa were hydrobid gastropods and the errant polychaete *Hediste diversicolor*, which were important for the within-group variability in, especially, the disturbed treatment (D). The average within-group dissimilarity was largest in the disturbed treatment (D, 77%), and smallest in the Elevated treatment (E, 20%).

### Relationship between macrofauna and nutrient fluxes - and the contribution of bivalves

Dark chambers were used to measure the net flux of oxygen and nutrients across the sediment-water interface in the absence of primary production.

**Figure 3:**
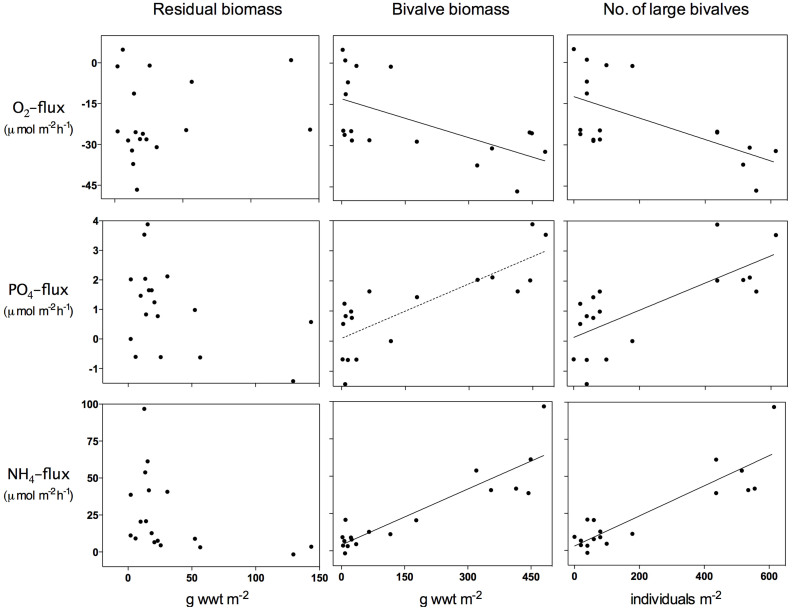
Relationships between residual biomass (total community biomass less bivalve biomass), bivalve biomass and the number of large bivalves (> 5 mm), and community respiration (O_2_) and nutrient (NH_4_^+^ and PO_4_^3−^) fluxes. Lines indicate a significant relationhip.

Bivalves were the dominant drivers of community biomass patterns and explained 98% of biomass variability (r^2^ = 0.98; p < 0.0001, linear regression) and were hence expected to drive ecosystem function relationships. Indeed, bivalve biomass and the number of large bivalves explained more of the variability in O_2_ consumption, NH_4_^+^ and PO_4_^3−^-fluxes than total residual community biomass (Fig. 3, Table 3). PO_4_^3−^-fluxes were exceedingly low, as is typical for sandy sediments low in organic matter.

In DistLM marginal tests, solute fluxes were correlated most strongly with the number of adult bivalves (Table 3). The only other significant predictors were the number of juvenile bivalves and number of species, which were weakly correlated with O_2_ flux. However in partial tests (i.e. after correcting for the influence of large bivalves) neither of these two variables was significant. The number of large bivalves was the best linear predictor of PO_4_^3−^ and NH_4_^+^ flux, explaining 53 and 79% of the variability, respectively. Including other variables in the model only explained an additional 3–5% of the variation. For the O_2_ flux, a combination of adult and juvenile bivalves and the number of species explained 19% more of the variation (cumulative r^2^ = 0.56) than a model containing just the number of adult bivalves. Interestingly, total community abundance had no significant effect on these fluxes.

## Discussion

We have demonstrated that intraspecific variations in body-size can be a key predictor of ecosystem functioning. We used a density manipulation experiment to explore the contribution of large deep-burrowing bivalves to oxygen and nutrient fluxes across the sediment-water interface, important measures of ecosystem function. We defaunated the seafloor a year in advance to initiate the community assembly process and observed recovery in terms of species-abundance distribution, but as expected observed only very limited recovery of macrofaunal biomass (Fig. 2, Table 2). Important members of the benthic community, such as adult polychaetes and gastropods colonized the disturbed plots, and juvenile bivalves were also observed in high numbers, but adult bivalves remained more or less absent (Table 1). To increase density variations in our experiment, we also added adult bivalves to undisturbed plots, and showed that bivalves dominate measures of ecosystem function. While the Elevated treatment (E) made an important contribution to the observed response, the bivalve densities in these plots were still within the natural density variation observed in the area. Importantly, we also observed distinct differences between disturbed and control plots (D vs. C), and the distribution of biomass across treatments formed a clear gradient (Fig. 2b). In contrast, species numbers and abundances were not significantly different between treatments.

An important goal in ecology, and for successful restoration and conservation, is to understand how species contribute to ecosystem processes, such as the rate and stability of nutrient cycling^1^. In soft-sediment habitats, shifts in ecosystem performance are often associated with changes in species influencing organic matter recycling and nutrient regeneration^22^,^39^,^40^. We used *in situ* flux chambers to examine the role of large individuals and their contribution to community respiration and sediment nutrient fluxes. In the analysis we ignored the categorical treatments and simply explored the relationship between biomass and large individuals and measures of ecosystem function across treatments (Fig. 3). Again, neither species diversity nor abundance could explain much of the variability in the observed responses. Our results show that the number of large bivalves were the strongest predictors of ecosystem function (Fig. 3). These results support earlier studies, reporting that presence of bivalves enhances benthic respiration and the release of ammonium through bioturbation and excretion^23^,^26^. Generally bioturbation can enhance the amount of fresh organic material transported into the sediment^40^, and stimulate microbial and meiofaunal activity, thus promoting organic matter degradation rates^41^,^42^ and the production and transportation of NH_4_^+^ and PO_4_^3−^ to overlying waters^43^. Particularly NH_4_^+^ fluxes are, however, in addition to sediment reworking also due to bivalve excretion^23^,^26^,^44^. Still, species-specific traits are likely to affect sediment redox-dependent processes in different ways and result in complex biogeochemical interactions. For example, both *Macoma* and *Mya* are sediment biodiffusers. The more shallow-burrowing *Macoma* is positioned in the sediment nitrification zone, and may enhance NO_3_^−^ efflux to the overlying water. In contrast, the deeper-dwelling *Mya arenaria*, transfers oxygen into the reduced zone of the sediment and may enhance nitrification-denitrification rates and thus cause an uptake of NO_3_^−^ 45. Hence animal-sediment interactions are complex and might result in different impacts on nutrient regeneration processes, depending on the biology and trait-composition expressed by the resident species. Nevertheless, our results unequivocally demonstrate that large bivalve individuals are strong predictors of ecosystem function. Although it would be intresting to conduct additional experiments where the same high biomass is made up of a large number of small indiviudals, such a situation is not likely to exist in natural bivalve beds. In addition, large bivalves often bury deeper in the sediment and can be expected to displace more sediment, pump more water and create stronger pore-water pressure gradients^17^.

Our study is one of the first to partition the contribution of large individuals to important measures of ecosystem function through an *in situ* manipulation of a real community. In fact, by manipulating bivalve size structure through the removal and addition of large individuals, we held species identity more or less constant, but altered the trait characteristics and functional diversity of the community^9^. Other taxa (e.g. polychaetes) at our study site are more fast growing than bivalves and were able to attain normal biomasses over the one-year recovery period. Our results highlight that without the presence of large adults, ecosystem functionality is radically changed. Bivalve species such as *Macoma balthica* and *Mya arenaria* have life-spans of 6–10 and 10–20 years, respectively, and maximum life-spans of 30 years have been reported for both species^46^,^47^. This indicates that while the regional supply of bivalve larvae and post-settlement juveniles may result in rapid colonization into disturbed habitat patches^36^,^48^, mature stages will take years to recover, especially since adult infaunal bivalves have limited mobility and recovery is thus largely dependent on individual growth. Inter- and intra-specific traits such as longevity and large size are disproportionately affected by habitat loss and too frequent disturbance regimes^49^. Increases in disturbance-regimes are hence of particular concern as the community assembly processes may be interrupted before bivalves reach full size and are able to contribute to important ecosystem processes. Indeed, historical reconstructions have highlighted that losses of suspension-feeding bivalves have profoundly influenced food webs and ecosystem function^50^.

In soft-sediment systems the degradation of macrobenthic communities as a result of disturbance has been shown to result in the loss of deep-burrowing large taxa and is predicted to reduce bioturbation^31^. Eutrophication-induced hypoxia and anoxia has spread widely across the world^51^ and is particularly common in the Baltic Sea in both coastal and open-sea waters^52^. The consequent loss of deep-burrowing and bioturbating taxa, and particularly their adult life-stages is of concern because of their major influence on all oxygen-dependent biogeochemical processes^41^,^43^. As highlighted by Ellison et al. (2005)^16^, the dynamics of communities shaped by foundation species, such as the bivalves in our system, may be dominated by a small number of strong interactions, which makes these types of communities fragile to switching between alternative stable states. In such communities disturbances have the potential to flip the ecosystem across a threshold into a different stability domain, and the probability for this to happen increases as foundation species are driven to regional functional extinction^53^.

Changes in biodiversity affect ecosystem functioning and can thus disrupt the way ecosystems contribute to valuable ecosystem services (e.g. nutrient regeneration processes^2^). Biodiversity losses typically involve declines in both abundance and biomass of common species, thus shifting dominance patterns of communities^1^,^54^. BEF studies have, however, mostly focused on species richness effects even though reported species identity or “sampling” effects are common, indicating that particular dominant traits may be underpinning ecosystem function^6^,^9^,^54^. We show that individual body-size is important for ecosystem functionality. As highlighted by Bengtsson (1998)^55^, body-size distributions have mechanistic links to ecosystem functions (e.g. energy flow and nutrient cycling), because most rates of ecosystem processes are mechanistically related to biomass through uptake, feeding and physiology. In contrast, the mechanistic link between species diversity and process rates is less clear. Our *in situ* findings support hypotheses put forward^9^,^10^ and experimental findings^20^ that evaluation of body size, not only between but also within species, provides important insights into the mechanisms behind biodiversity effects on ecosystem function. Importantly, we show that large individuals in natural communities may have a major influence on ecosystem function. If local disturbances are recurrent, preventing individuals from reaching large sizes, the contribution of large adult stages may be lost, with severe implications for ecosystem functionality^34^. The characterization of body size and its importance for communities and ecosystem function has ramifications for conservation and restoration efforts, because it facilitates the interpretation of how disturbances, through the functional elimination of species (i.e. the loss of large-sized individuals), might propagate through the ecosystem^56^.

## Methods

### Study area

There are few areas where the loss or degradation of both habitat and species diversity is as evident as in the Baltic Sea. Hypoxic zones cover up to 70.000 km^2^ that are largely devoid of all benthic macrofauna^52^,^57^ and it is clear that the reduction in the distribution and diversity of Baltic Sea benthos due to hypoxic events has already altered the way benthic ecosystems contribute to key ecosystem processes (i.e. nutrient cycling). Recently, the problem of seasonal hypoxia in shallower, near-shore areas has also been highlighted^37^,^51^,^52^. Our experiment was conducted in the northern Baltic Sea, near Tvärminne Zoological Station (59° 50′ 44″ N, 23° 14′ 96″ E), Finland. The site was at 4 m depth and had sandy sediments, with a median sediment grain size of 0.29 mm, organic matter content of 0.5 ± 0.03% (SD), and total carbon- and nitrogen content of 0.18 ± 0.02 and 0.02 ± 0.01%, respectively. Salinity is around 6 and there are no tides in the area.

### Experimental setup

The experiment included three treatments; the disturbed (D), the control (C) and one treatment with an elevated number of adult bivalves (E). One year before the flux measurements, three blocks were established along a 50 m transect line. For the disturbed treatment, a 16 m^2^ plot was defaunated in each block by covering the sediment surface with black LDPE plastic to induce anoxia to underlying sediments. The disturbance manipulation simulated patchy hypoxia, for example induced naturally by drifting algal mats^37^. Plots were covered for a period of 16 days, to ensure complete defaunation and the plastic was removed in late July 2006 (details in^36^). Following the experimental manipulation, large dead bivalves were observed to have emerged to the sediment surface, a common escape response to hypoxia (Fig. 1). One year later, the control and elevated treatments plots were placed > 2 m from the disturbed plots on unaffected sediments. To the elevated treatment, 20 large (>10 mm) *Macoma balthica* and one large (> 30 mm) *Mya arenaria* were added, corresponding to natural densities in the area. Historical data confirm that large *Macoma* (>10 mm) occurred in such densities (i.e. ≥ 396 ind. m^−2^) in our study area during the 1920s and 1930s^58^, when conditions were undisturbed by eutrophication. All manipulations and sampling were done using SCUBA. To include variations in environmental conditions and to increase the generality of our results, we measured the selected ecosystem functions on two different occasions, in mid-August (T1) and in mid-September 2007 (T2).

### Measurements of sediment nutrient and oxygen fluxes

Measurements of sediment nutrient and oxygen fluxes were performed with one dark benthic chamber (504 cm^2^, volume 6 l) for each treatment replicate (i.e. 18 chambers in total; see^43^ for methodological details). Chamber frames were deployed (pushed 6.5 cm into the sediment) one day before the incubation started. Simultaneously, large bivalves were added to the elevated treatment and allowed to rebury over night, with nets keeping potential predators from foraging on the bivalves. Incubation started once chamber lids were installed and ended 6 hours later. Water samples were taken from the chambers at start and end of the incubation. The chamber water was manually stirred with a paddle from the outside before water samples were withdrawn with syringes (in total 200 ml) using a sampling port in the chamber lid. The initial water volume of the tube was discarded, before the chamber water was collected. Replacement water was supplied through a port that was placed distant from the sampling port. To correct for water column effects, three dark 1 l LPDE bottles were used for incubation of ambient water at 4 m depth during the experiment.

Water samples were processed immediately on the boat. For determination of dissolved oxygen, 60 ml of each sample was fixed with 0.5 ml Mn(OH)_2_ and 0.5 ml KI. The rest of each sample was filtered through a Whatman GF/F filter, directly into a 250 ml Nalgene bottle for nutrient analyses. All samples were stored on ice in the dark during transport to the laboratory and nutrient samples were then frozen (−20°C) until further analysis. Dissolved oxygen concentrations were determined by the Winkler procedure, while NH_4_^+^ and PO_4_^3−^ were measured with Lachat flow injection analysis.

### Sampling of benthic fauna and sediment

Samples for sediment organic matter, total carbon (TC) and total nitrogen (TN) were taken with a 2.1 cm diameter core from the control and disturbed sediments. In the laboratory, the surface sediment (upper 1 cm) was stored at –20°C until analysis. Sediment organic matter was determined by loss on ignition (3 h at 500°C). Sediment for nutrient analyses was freeze-dried and thoroughly homogenized. Analyses of TC and TN in sediments were performed with a Carlo Erba high temperature combustion elemental analyzer.

After the incubation, chamber lids were removed and one macrofaunal core (Ø 5.6 cm, depth 15 cm) sample was taken from the middle of each chamber. The area enclosed by each chamber was then excavated (to 30 cm depth), in order to account for any deeper-burrowing bivalves. Large excavated bivalves (> 5 mm) were counted and measured (shell length and wet weight, including shell).

The macrofaunal core samples were preserved in 70% ethanol and stained with Rose Bengal. To account for small recruits, the samples were elutriated by first suspending the sediment in a bucket of spinning water and decanting off the supernatant through a 200 μm sieve (repeated five times), and then checking the remaining sediment for any larger animals. The fauna was identified, counted and measured at 10 × magnification. To obtain the proportions of juveniles and adults of dominant taxa, shell lengths of bivalves and gastropods, and the width of the 10th setiger of *Hediste diversicolor* and *Marenzelleria* sp. were measured. Gastropods < 1 mm shell length were only identified to family. The total weight of each species was determined (precision 0.1 mg blotted wet weight, including shell).

### Data analysis

Multivariate analyses of benthic community data were performed with the PRIMER software^59^. Multidimensional scaling (MDS) and PERMANOVA were used to identify differences in abundance and biomass between treatments, while the SIMPER analysis was used to identify species contributions to (dis)similarities within and between treatments. Bray-Curtis similarity coefficient was based on untransformed data. Distance-based linear models (DistLMs) in PERMANOVA+^59^ were used to determine if macrofauna variables were significant predictors of O_2_ consumption, PO_4_^3−^ and NH_4_^+^ fluxes. Predictor variables included the abundance of juvenile (< 5 mm shell length) and adult bivalves, residual community abundance and biomass (i.e. less the contribution of bivalves) and the number of species. Marginal tests were run to identify strong, significant predictors irrespective of other variables, then partial tests were performed to assess the explanatory value of a predictor variable after other variables had been accounted for. The number of large bivalves and bivalve biomass were strongly collinear and therefore only the former was included. Remaining predictor variables were weakly correlated with each other (Pearson's r < 0.4). Similarity matrices of untransformed fluxes were constructed using Euclidean distances and the model was run using the step-wise selection procedure and R^2^ selection criterion. P values were obtained for predictor variables by 99999 permutations.

## Figures and Tables

**Table 1:** Species numbers, total community and bivalve abundances and biomasses across treatments

	Disturbed		Control		Elevated	
Variables	ave	SE	ave	SE	ave	SE
Maximum no of spp.	11		11		10	
Average no of spp.	8.7	0.4	8.8	0.3	8.8	0.3
Community abundance (ind. m^−2^)	27564.3	1769.5	20568.3	1463.9	20816.2	2161.5
Bivalves abundance	4975.5	944.5	3030.3	602.2	3593.9	609.0
*Macoma balthica*	4272.3	814.2	2606.1	498.5	3146.6	553.7
*Mya arenaria*	71.8	47.1	3.3	2.2	96.6	46.6
Community biomass (g wwt m^−2^)	56.2	12.9	97.8	17.6	458.1	39.4
Bivalve biomass	11.0	2.2	81.8	18.1	440.8	39.8
*Macoma balthica*	8.2	2.1	58.6	13.8	296.9	19.6
*Mya arenaria*	0.1	0.0	21.0	14.6	143.2	38.5

**Table 2:** Results from the multivariate permutational analysis (PERMANOVA) of differences in total abundance and biomass between treatments. C = control, D = disturbed, E = elevated

PERMANOVA	df	SS	MS	Pseudo-F	P(perm)
Abundance					
Treatment	2	2692	1346.1	1.525	0.126
Residual	33	29126	882.6		
Total	35	31818			

Biomass	df	SS	MS	Pseudo-F	P(perm)
Treatment	2	45975	22987.0	14.677	0.001
Residuals	33	51685	1566.2		
Total	35	97660			
Pair-wise test	t	P(perm)			
D-C	2.276	0.001			
D-E	5.135	0.001			
C-E	4.302	0.001			

**Table 3:** Correlation coefficients between macrofauna variables and solute fluxes derived from DistLMs. Marginal tests examine a single predictor separately, while partial tests take into account the effect of the remaining predictors. Residual refers to macrofauna community parameters less the contribution of adult and juvenile bivalves

	O_2_-flux		PO_4_-flux		NH_4_-flux	
Variables	Marginal	Partial	Marginal	Partial	Marginal	Partial
# adult bivalves	0.61**	0.47*	0.73***	0.54**	0.89***	0.71***
# juvenile bivalves	0.47*	0.25	0.24	0.07	0.15	0.03
Residual abundance	0.04	0.03	0.10	0.09	0.16	0.14
Residual biomass	0.19	0.14	0.43	0.16	0.41	0.17
# species	0.48*	0.26	0.32	0.07	0.21	0.09

*p < 0.05;

**p < 0.01;

***p < 0.001.
